# An Evaluation of Growth Hormone and IGF-1 Responses in Neonates with Hyperinsulinaemic Hypoglycaemia

**DOI:** 10.1155/2013/638257

**Published:** 2013-10-08

**Authors:** Senthil Senniappan, Khalid Hussain

**Affiliations:** ^1^Department of Paediatric Endocrinology, Great Ormond Street Hospital for Children, NHS Trust, WC1N 3JH, UK; ^2^Developmental Endocrinology Research Group, Molecular Genetics Unit, Institute of Child Health, University College London, 30 Guilford Street, London WC1N 1EH, UK

## Abstract

*Background*. Hyperinsulinaemic Hypoglycaemia (HH) is the most common cause of severe and persistent hypoglycemia in the neonatal period. It has been shown that the neonates with HH fail to generate adequate serum cortisol counterregulatory response to symptomatic hypoglycemia. However the role played by growth hormone (GH) and Insulin-like Growth Factor 1 (IGF-1) is not clear. *Objectives*. To compare the serum GH, IGF-1, and IGFBP3 responses to HH in neonates undergoing diagnostic fasting studies. *Population and Methods*. Data was retrospectively collected on full-term neonates who presented with severe and persistent hypoglycemia and were confirmed to have HH. Neonates born with intrauterine growth retardation or those on medical therapy (diazoxide or octreotide) were excluded. *Results*. 31 neonates with HH (mean gestational age: 38 weeks and mean birth weight: 3.9 kg) were included in the study. The mean age at the time of diagnostic fast was 4 weeks, the mean glucose concentration during the fast was 2.2 mmol/L (SEM ± 0.09), and the mean insulin level was 11.9 mU/L (±2.12). The mean serum GH concentration during the hypoglycaemia was 12.5 *µ*g/L (±1.53). The mean serum IGF-1 and Insulin-like Growth Factor Binding Protein 3 (IGFBP3) levels were 29.2 ng/ml (±7.8) and 1.21 mg/L (±0.13), respectively. The mean cortisol concentration was 201 nmol/L (±33). *Conclusions*. Whilst the serum IGF-1 and IGFBP3 levels are relatively low during hypoglycaemia, the serum GH level does reflect an appropriate counterregulatory response to HH. The serum cortisol counterregulatory hormonal responses are blunted. Further studies are required to understand the mechanism(s) of these hormonal alterations in neonates with HH.

## 1. Introduction

Hyperinsulinaemic Hypoglycaemia (HH) is the most common cause of recurrent and persistent hypoglycemia in the neonatal period [1]. It is characterized by the inappropriate and excessive secretion of insulin from the pancreatic *β*-cells in relation to the blood glucose concentration. It is a heterogeneous disease wherein patients present typically with persistent hypoketotic hypofattyacidaemic hypoglycaemia [2]. The biochemical abnormalities reflect the metabolic actions of insulin, causing inhibition of glucose production (glycogenolysis and gluconeogenesis), inhibition of lipolysis, and ketone body formation with simultaneous increase in glucose utilization [3]. 

The molecular basis of the most common forms of HH involves mutations in the genes *ABCC8* and *KCNJ11* encoding the two subunits SUR1 and KIR6.2, respectively, of the pancreatic *β*-cell ATP-sensitive K^+^ channel (K_ATP_ channel) [4]. The K_ATP_ channel plays a crucial role in glucose homeostasis by linking glucose metabolism to electrical excitability and insulin secretion [5]. The other causes include mutations in *GLUD1, GCK, HADH, SLC16A1, HNF4A*, *HNF1A*, and *UCP2 *genes that lead to hypersecretion of insulin from the *β*-cells dependent or independent of nutrient stimulation [6, 7]. 

The counterregulatory hormones play an essential role in the maintenance of normal blood glucose concentration. Cortisol stimulates gluconeogenesis and antagonizes the actions of insulin. Glucagon, epinephrine, and norepinephrine form a primary defense against hypoglycemia. The secretion of GH from pituitary somatotrophs is controlled by the complex interaction between two neuropeptides, growth hormone releasing hormone (GHRH) and somatostatin (SS). It is thought that pulses of GHRH are secreted into the hypophysial portal blood during troughs of SS release in rodents [8], but the main determinant of pulsatile GH release, as shown in studies in sheep, is the GHRH pulse occurring at the times of a nadir in SS release [9]. 

GH has an important role in postnatal somatic growth by stimulating the production of Insulin-like Growth Factor 1 (IGF-1) in the liver and target organs. Whilst pituitary-derived GH is not essential for fetal growth, both IGF-1 and IGF-2 are involved in cell growth and differentiation during the fetal period [10]. IGFs do not cross the placenta, and during fetal development they are synthesized in a GH-independent fashion [10]. Besides GH, the nutritional status of the cell is a potent regulator of IGF-1 serum levels. After birth, IGF-1 concentrations rise in response to GH and increase steadily throughout childhood [11]. IGF-1 concentrations are influenced by age, puberty, and nutritional status. Certain disease states may increase IGFBP (IGF binding proteins) proteases with a consequent drop in circulating IGF-1 [12]. Serum Insulin-like Growth Factor Binding Protein 1 (IGFBP-1) level is high at birth [10]. Insulin impairs IGFBP-1 gene expression, and, as a consequence, high levels of IGFBP-1 are present in diabetes mellitus [10] and low levels are noted during hypoglycaemia in patients with congenital hyperinsulinism [13].

The precise role played by the various counter regulatory hormones such as cortisol, GH, epinephrine, norepinephrine, and glucagon in HH is not fully understood. Neonates with HH have been shown to fail to generate an adequate serum cortisol counter regulatory response [14]. This has been shown to be due to the lack of drive from the hypothalamic-pituitary axis, with inappropriately low plasma ACTH concentrations at the time of hypoglycemia [15]. It has also been shown that children with HH display blunted serum glucagon counter regulation with normal epinephrine and norepinephrine responses [16]. In this study, we have assessed the responses of serum GH, IGF-1, IGFBP3, and cortisol during hypoglycaemia in neonates undergoing diagnostic fasting studies.

## 2. Materials and Methods

Retrospective data on the hormone responses was collected on 31 full-term neonates who presented with severe and persistent hypoglycemia and were confirmed biochemically to have HH. Neonates born with intrauterine growth retardation or with associated syndromes or those on medical therapy (diazoxide or octreotide) were excluded. Each of these patients was referred to a tertiary referral center for investigation of their hypoglycemia. None of the patients were hypoglycemic 48 hours before the beginning of the study. Normoglycaemia (4–8 mmol/L) was maintained in the neonates with HH by continuous intravenous infusion of dextrose using central venous catheters. The diagnostic work-up for the hypoglycemia involved carrying out a controlled fast that involved stopping all enteral feeds and intravenous fluids. Blood glucose concentrations were measured hourly during the fast and more frequently if the patient was becoming hypoglycaemic. The fast was completed when the plasma glucose concentration fell to <3 mmol/L or if the child became symptomatic. 

At the time of hypoglycemia, each child had blood withdrawn for measurement of blood glucose, serum insulin, GH, IGF-1, IGFBP3, cortisol, plasma nonesterified fatty acids (NEFAs), and 3-hydroxybutyrate, through an indwelling intravenous catheter. The hypoglycemic event was then treated with intravenous fluids. The diagnosis of HH was made biochemically by the typical finding of HH with hypofattyacidemia and hypoketonemia and a raised glucose infusion rate (>8 mg/kg/min) required to maintain normoglycaemia [1].  Table 1 shows the characteristics of the patient group. The results are expressed as mean serum hormone concentrations (± 1SEM).

## 3. Hormone Assays

Serum insulin, cortisol, GH, IGF-1, and IGFBP3 were measured using the immunometric assay with the Siemens Immulite 2500 Analyser. The coefficients of variation (CV) for GH and IGF-1 were between 2.9–4.6% and 2.3–3.9% for intraassay and between 5.5–6.6% and 3.7–8.1% for the inter-assay, respectively. The CV for IGFBP3 for the intraassay was between 4.1 and 4.8% and that for the inter-assay was between 5.2 and 7.3%. The CV for cortisol for the intraassay was between 5.1 and 7.4% and that for the inter-assay was between 6.8 and 9.4%. The plasma concentrations of NEFA and ketone bodies were determined by colorimetric spectrophotometry using the ILab 650 Analyser. The plasma glucose was measured by dry-slide colorimetric methods using Ortho Vitros 5600 Analyser (CV 1.3%). 

## 4. Results

**Figure 1:**
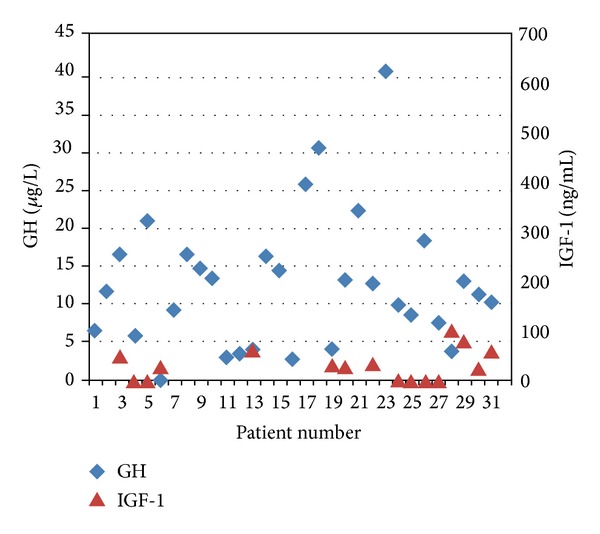
Graph shows rise in GH concentrations whilst the serum IGF-1 levels are low during the episodes of hypoglycaemia in patients with HH.

The mean concentrations of the counter regulatory hormones (cortisol, GH) and intermediary metabolite results are shown in Table 2. Consistent with the clinical diagnoses, the neonates with HH showed excessive and inappropriate insulin release during hypoglycemia. The serum IGF-1 and IGFBP3 levels were available only in 16 out of 31 patients. The serum GH concentration at the time of hypoglycemia was noted to rise to a mean level of 12.5 *μ*g/liter whilst the mean IGF-1 and IGFBP3 concentrations were low at 29.2 ng/mL and 1.21 mg/L, respectively. Around 50% of babies had undetectable IGF-1 level. The mean cortisol concentration was noted to be low (201 nmol/L) during the episode of hypoglycaemia. GH concentrations are compared with serum IGF-1 levels in Figure 1. 

## 5. Discussion

GH and cortisol play an essential role in glucose counter regulation after an episode of hypoglycemia [18]. GH and cortisol have numerous effects on glucose metabolism, including increasing the rate of gluconeogenesis and antagonizing the effects of insulin. In adults, the glycemic thresholds for the activation of glucose counter regulatory hormones such as GH and cortisol lie within or just below the physiological blood glucose concentration and slightly higher than the threshold for symptoms [19]. This implies that GH and cortisol secretion increase in response to blood glucose concentrations within the normoglycaemia range, and it is thought that these increases are inversely proportional to the nadir in blood glucose [20]. 

It has been shown that during the newborn period, serum GH levels are higher than in childhood [21, 22]. In a study involving 36 healthy appropriate-for-gestational-age (AGA) newborns, the mean GH serum level was noted to be 29 *μ*g/liter (SD ± 17) at the age of 3 days [23]. Similarly a mean GH serum level of 24.2 *μ*g/liter (SD ± 12.3) was observed in 30 healthy AGA newborns at the age of 3 days [24, 25]. In a study using 314 neonatal blood spot samples for GH assessment, it was shown that females had significantly higher GH values than males (mean 17.5 versus 15.4 *μ*g/liter, *P* = 0.0137) [25]. However no effect of birth weight, birth length, head circumference, duration of gestation, mode of delivery, Apgar score, or preeclampsia was noticed on GH serum concentrations [25]. We observed appropriate elevation of GH in our group of patients with HH possibly as a counter regulatory response to hypoglycaemia although, the GH levels are slightly lower than those shown in the other studies, possibly due to the age differences of the infants [23–25]. 

Previous studies have identified a lack of serum GH response to spontaneous hypoglycemia [26, 27]. It has been suggested that the inappropriately low serum GH response observed in spontaneous hypoglycemia could be due to pulsatile secretion of GH with a short half-life (10 minutes) [26]. In a study comparing the GH response of a group of children with spontaneous hypoglycemia against a group of children with insulin induced hypoglycaemia (as part of insulin tolerance test, ITT), it was noted that GH response was significantly lower in children with spontaneous hypoglycaemia [26]. It was suggested that rapid fall of blood glucose in ITT group could have been a factor in better GH response as opposed to the group with spontaneous hypoglycaemia [26]. However studies in normal and diabetic adults showed that the rate of fall in the blood glucose concentration does not affect the counter regulatory responses to hypoglycemia and that the counterregulatory hormone response to hypoglycemia was triggered by the glucose level per se [28]. 

IGF-1 is an important growth factor during intrauterine life. A strong association between umbilical cord serum concentrations of IGF-1 and birth size has been observed in several studies [29, 30]. In a study involving 153 delivering mothers and their offspring at birth, association between serum concentrations of ghrelin, leptin, insulin, IGF-1, and IGFBP-3 were evaluated [17]. It was shown that the babies with intrauterine growth retardation showed relatively increased GH and low IGF-1 and IGFBP-3 concentrations, relatively low leptin, and increased ghrelin values [17]. It has been postulated that fetal growth in an environment of relative nutrient deprivation induces this hormonal adaptation [17]. In large-for-gestational-age (LGA) babies, leptin, IGFBP-3, insulin, and glucose concentrations were noted to be significantly higher in asymmetric LGA newborns than in symmetric LGA and AGA newborns [17]. In a study involving 26 babies at a median of 4.5 days of age, IGF-1 levels were higher in appropriate-for-gestational-age babies (AGA; mean ± SD, 82 ± 61 ng/mL) than in small-for-gestational-age (SGA; 34 ± 22 ng/mL) babies [31] whilst the baseline, mean, and peak GH levels were higher in SGA babies than in AGA babies. IGF-1 levels in our cohort (mean, 29.2 ng/mL) are lower than that expected for AGA babies and are in comparison with the levels seen in SGA babies. Given that the mean birth weight of our cohort is in the normal range, the lower IGF-1 levels appear to be independent of the birth weight and possibly a specific effect of hyperinsulinism. However, it has to be noted that IGF-1 levels were available only in 16 out of 31 patients due the retrospective nature of the study. None of the mothers of the infants in our cohort were known to have gestational diabetes making any differences in body fat composition unlikely within the cohort.

In hyperinsulinaemic conditions, the mitogenic action of insulin is believed to be mediated by IGF-1, affecting the rate of transcription of the IGF-1 gene [10]. Given the structural homology between the insulin receptor and the IGF-1 receptor, insulin has been thought to interact with IGF-1 receptor exerting direct mitogenic effect and macrosomia [10]. On the contrary, we observed relative IGF-1 deficiency that raises the possibility of downregulation of the expression of GH receptor in the liver. It has been noted that nutrient intake can alter IGF-I levels acutely and a short period of starvation can reduce IGF-I [32]. Studies have indicated that GH-IGF-1 axis is closely related to feeding in the newborn [31]. Majority of our babies were on intravenous fluids with high glucose concentrations to maintain normoglycaemia. The enteral feeding was either reduced or stopped temporarily whilst stabilizing the blood glucose concentrations. This state of nutritional deprivation due to delayed enteral feeding could potentially have had some effect on IGF-1 levels in these babies. 

Studies on adults have shown that increases in plasma NEFA levels inhibit GH responses to a variety of pharmacological and physiological stimuli [33]. Pharmacological reductions in circulating NEFA cause GH release, and NEFA elevations reduce or block GH secretion stimulated by a variety of physiological or pharmacological conditions [34, 35]. It is thought that NEFA blocks GH secretion by acting directly at the level of the pituitary gland and blocks GHRH-stimulated GH secretion [33]. In our study plasma NEFA concentrations were suppressed with a mean value of 0.28 mmol/liter (range 0.05 to 1.42 mmol/liter), due to the dominant anabolic effects of insulin inhibiting the lipolytic response to hypoglycemia. 

In summary, the results from our study have clearly demonstrated that the serum GH concentrations in patients with HH do rise during the episode of hypoglycaemia whilst the IGF-1 levels are relatively low. The cortisol concentrations are low during the episode of hypoglycaemia as demonstrated in previous studies [15]. Further studies are necessary to understand the precise mechanism of GH and IGF-1 alterations in this group of patients. 

## Figures and Tables

**Table 1:** Patient characteristics.

Total number of patients	31	
Male : female	16 : 15	
Ethnicity		
Caucasian	29	
Asian	2	

	Mean	Range

Gestational age (weeks)	38	35–41
Birth weight (kg)	3.9	2.8–5.3
Age at diagnostic fast (days)	28	3–137

**Table 2:** Hormone responses in neonates with HH during the episode of hypoglycaemia.

	Mean	SEM (±)	Normal range
Glucose (mmol/L)	2.2	0.09	4–8 mmol/L
Insulin (mU/L)	11.9	2.12	<1.0 (during hypoglycaemia)
Growth hormone (*μ*g/L)	12.5	1.53	16.4–20.0*
IGF-1 (ng/mL)	29.2	7.8	90–110*
IGFBP3 (mg/L)	1.21	0.13	1.8–2.2*
Cortisol (nmol/L)	201	33	450
NEFA (mmol/L)	0.28	0.05	Depends on the duration of fast
3-betahydroxybutyrate (mmol/L)	0.05	0.003	Depends on the duration of fast

*Quoted from reference [17].
